# Diagnosis, treatment, and biosafety protection of imported highly pathogenic cutaneous fungal infection: a case-based study of cutaneous coccidioidomycosis

**DOI:** 10.3389/fpubh.2026.1833316

**Published:** 2026-05-12

**Authors:** Yuhua Weng, Zehu Liu, Xiujiao Xia

**Affiliations:** Department of Dermatology, Hangzhou Third People's Hospital, Hangzhou Third Hospital Affiliated to Zhejiang Chinese Medical University, Hangzhou, China

**Keywords:** biosafety precautions, *Coccidioides posadasii*, cutaneous, highly pathogenic microorganisms, non-endemic region, treatment

## Abstract

Coccidioidomycosis is a significant fungal disease primarily endemic to the southern United States, northern Mexico, and South America. Coccidioidomycosis is non-endemic in China, with only approximately 47 cases reported over the past 63 years. Recognizing that increased global travel is altering regional disease epidemiology, the Chinese Health Commission has classified *Coccidioides* species as highly pathogenic pathogens. The diagnosis of coccidioidomycosis poses a major challenge in China for medical institutions without biosafety level 3 (BSL-3) laboratories. In November 2021, we diagnosed a case of imported cutaneous coccidioidomycosis caused by *C. posadasii* from the United States. Shortly after returning from Arizona, USA, the patient presented with pneumonia-like symptoms accompanied by erythematous skin rash. The diagnosis was confirmed by next-generation sequencing (NGS), direct microscopy, histopathological examination and fungal culture. The patient had fatty liver disease with elevated liver enzymes. During treatment with oral fluconazole (400 mg daily), liver enzyme levels fluctuated persistently, and the patient exhibited intolerance to liposomal amphotericin B. Following proactive weight reduction and lipid-lowering therapy, along with intermittent administration of fluconazole, the patient's liver enzymes normalized. In October 2025, a 2-week course of liposomal amphotericin B led to clinical improvement, although direct microscopy remained positive. The patient is currently continuing fluconazole treatment. This case suggests that infections caused by highly pathogenic fungi in non-endemic regions may represent a major public health challenge globally, encompassing limitations in diagnostic tools, lack of treatment experience, and biosafety concerns.

## Introduction

In August 2023, the National Health Commission of China issued the Directory of Human Infectious Pathogens, which classifies seven fungi as highly pathogenic microorganisms under Risk Category 2. These fungi include *Blastomyces dermatitidis, Coccidioides immitis, Coccidioides posadasii, Histoplasma capsulatum*, other pathogenic species of the genus *Histoplasma, Paracoccidioides brasiliensis*, and other pathogenic species of the genus *Paracoccidioides*. Most of these pathogens are imported exotic fungi. According to the requirements of the Directory, live culture manipulation and animal infection experiments involving such highly pathogenic fungi must be conducted in Biosafety Level 3 laboratories (1). Culture and histopathological assessment are the definitive diagnostic methods for coccidioidomycosis (2). Therefore, the diagnosis of coccidioidomycosis poses a major challenge for medical institutions that lack Biosafety Level 3 (BSL-3) laboratories. In addition, for most patients requiring treatment for coccidioidomycosis, fluconazole is the first-line triazole agent (3).

**Figure 1:**
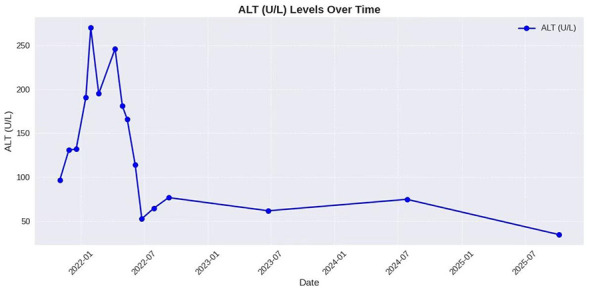
Changes in the patient's ALT levels over time during treatment.

In November 2021, we diagnosed a case of cutaneous coccidioidomycosis through next-generation sequencing (NGS), direct microscopy, histopathological examination and fungal culture. The patient was a student at the University of Arizona, USA, and presented with obesity, fatty liver disease, and elevated liver enzymes. The patient was initially treated with fluconazole (400 mg daily). During the course of treatment, liposomal amphotericin B was attempted but led to severe intolerance. Due to persistently elevated alanine aminotransferase (ALT) levels, fluconazole was administered intermittently. Following weight reduction and lipid-lowering therapy, the patient's ALT returned to normal (Figure 1). In October 2025, the patient received a 2-week course of liposomal amphotericin B, which resulted in clinical improvement. Currently, the patient continues to receive fluconazole therapy. We present this case of refractory cutaneous coccidioidomycosis to share our experience in its management, diagnosis, and biosafety precautions. This case study has been reported in line with the CARE guidelines (4), and the study was approved by the Medical Ethics Committee of Hangzhou Third People's Hospital (No.2026KA076).

### Patient information

On November 1, 2021, a 22-year-old male was admitted to the hospital with a five-month history of erythematous papules and swelling on his right arm and back. The patient was a student at the University of Arizona in the United States. Approximately 6 months prior, around 1 week after returning to China, he developed fever accompanied by extensive rash, primarily on his back. He sought treatment at another tertiary grade a hospital in Hangzhou, where a provisional diagnosis of pneumonia of unknown origin was made. Initial antibacterial therapy was administered for 2 weeks but yielded no significant improvement. Subsequently, intravenous fluconazole was given empirically for another 2 weeks, after which his fever subsided markedly, and he was discharged. Following discharge, the rash continued to expand and was associated with mild itching and pain, leading him to seek further consultation in the dermatology department of our hospital.

**Figure 2:**
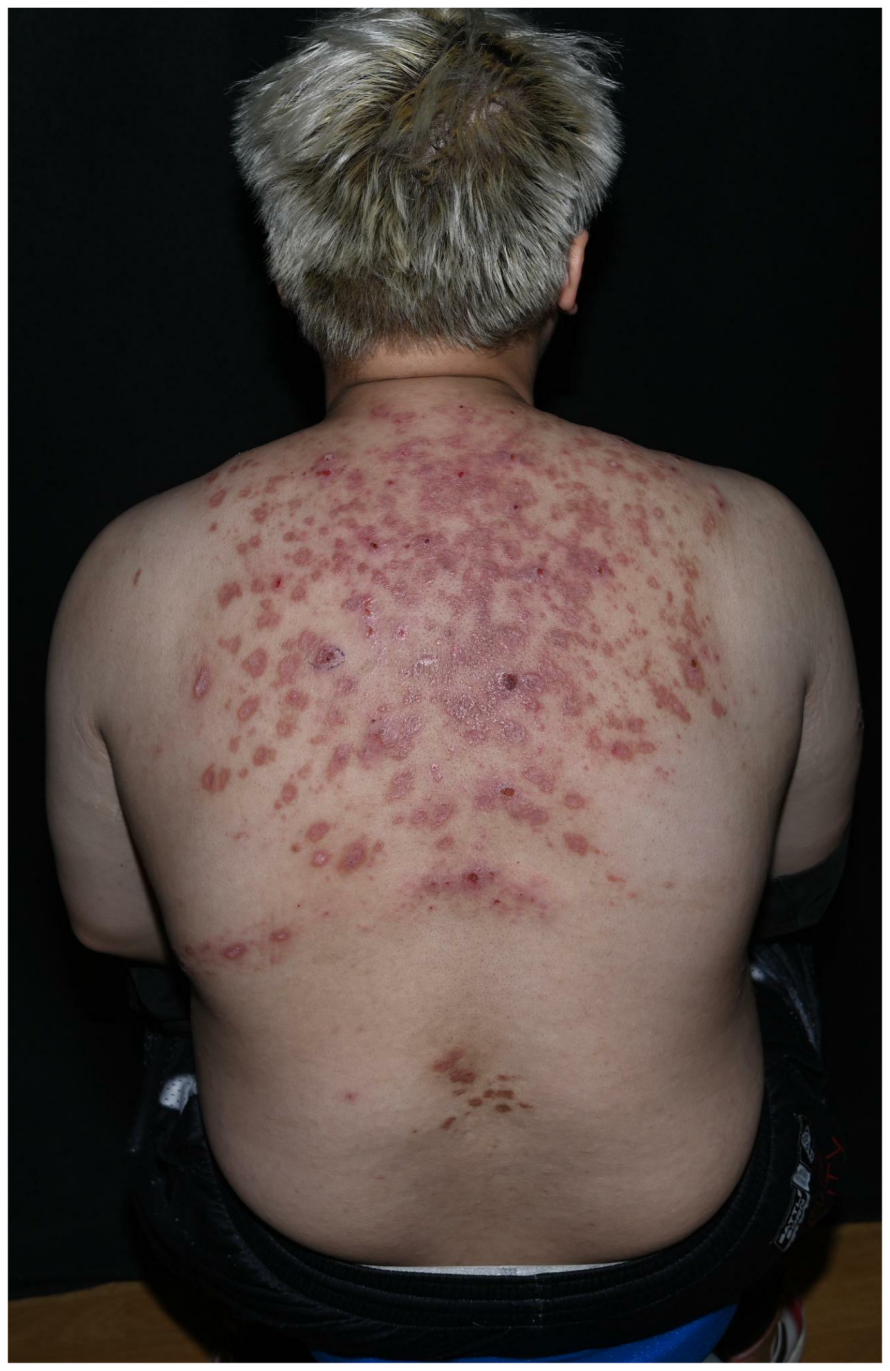
On the patient's back, dense patches of erythema and papules are observed, some of which have coalesced to form larger plaques with central depression.

Dermatological examination revealed erythema and papules on the skin of the right upper arm, with mild swelling. Scaling and excoriations were observed on the surface of the erythematous papules, along with a small amount of blood crusting. The back showed scattered, dense patches of erythema and papules, some of which had coalesced into larger plaques with central depression (Figure 2). Laboratory tests revealed an alanine aminotransferase (ALT) level of 97 U/L (normal range: 9–50 U/L), a homocysteine level of 44.9 μmol/L (normal range: 5.0–15.0 μmol/L), and a uric acid level of 558 μmol/L (normal range: 208–428 μmol/L). Abdominal ultrasound indicated fatty liver. Chest CT showed a solid nodular shadow in the lower lobe of the left lung.

**Figure 3:**
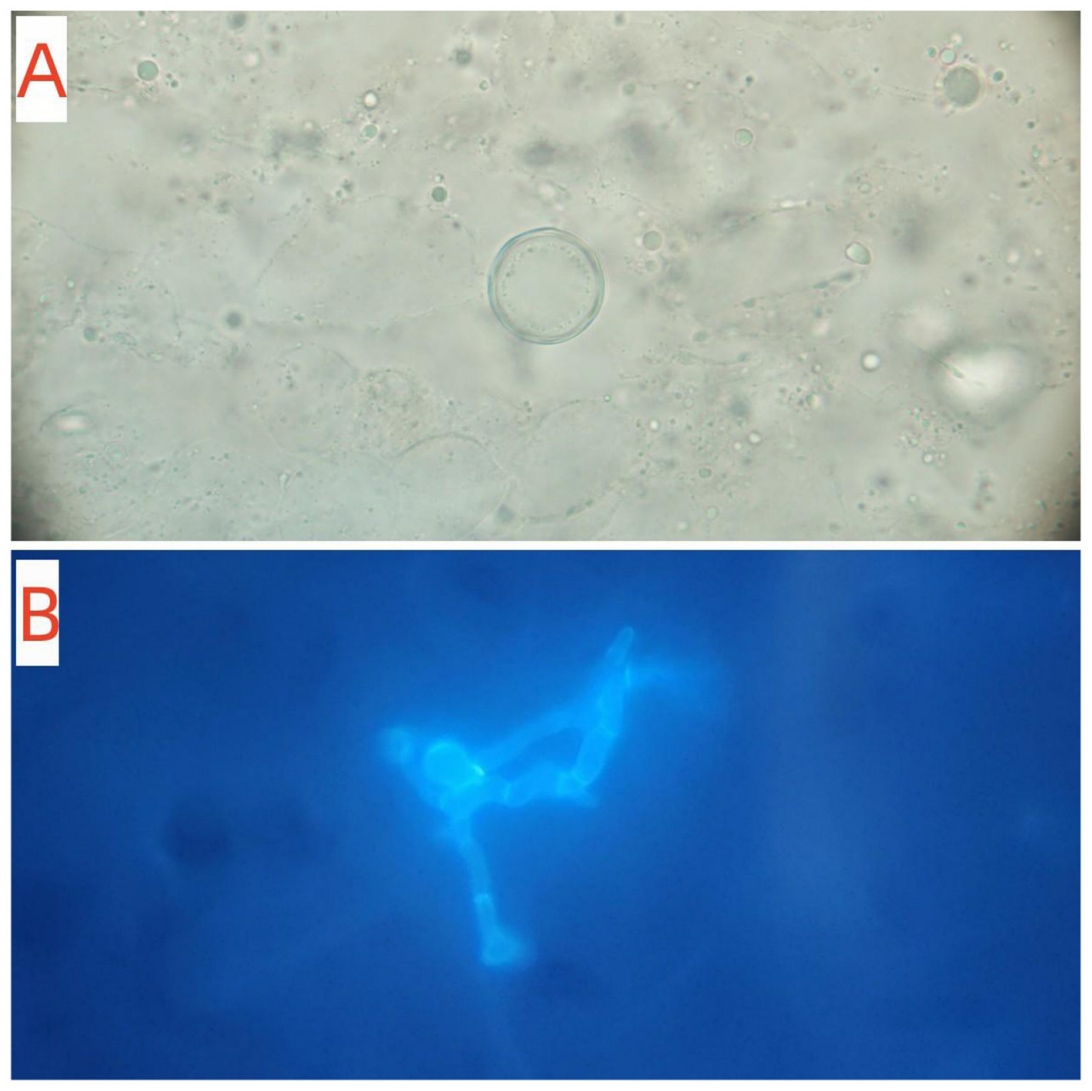
Direct microscopic examination of the scrapings reveals Coccidioidal spherule (**A**, 10% KOH × 1,000) and hyphae (**B**, calcofluor white stain × 400).

**Figure 4:**
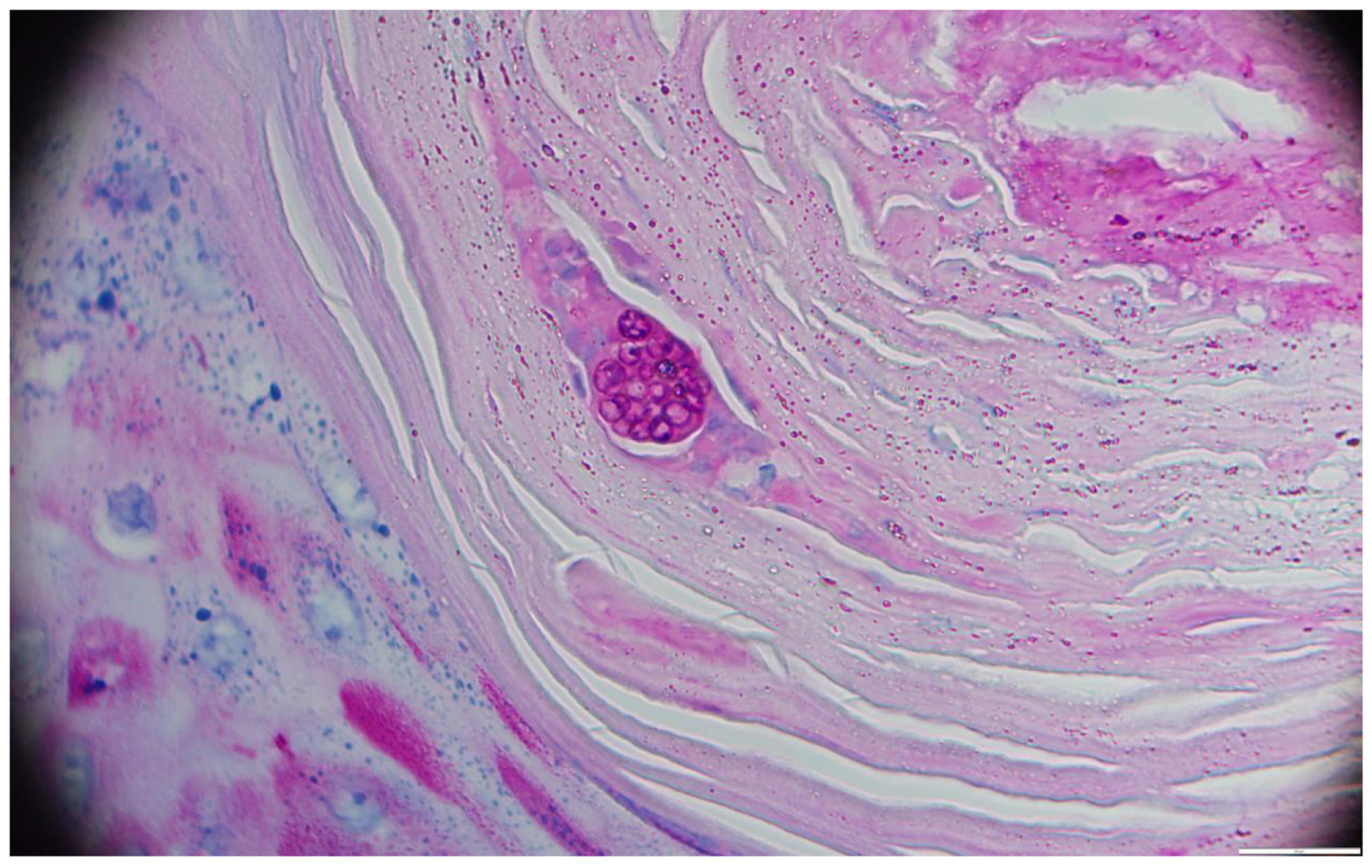
Histological examination reveals a spherule with endospores within a giant cell (PAS × 1,000).

**Figure 5:**
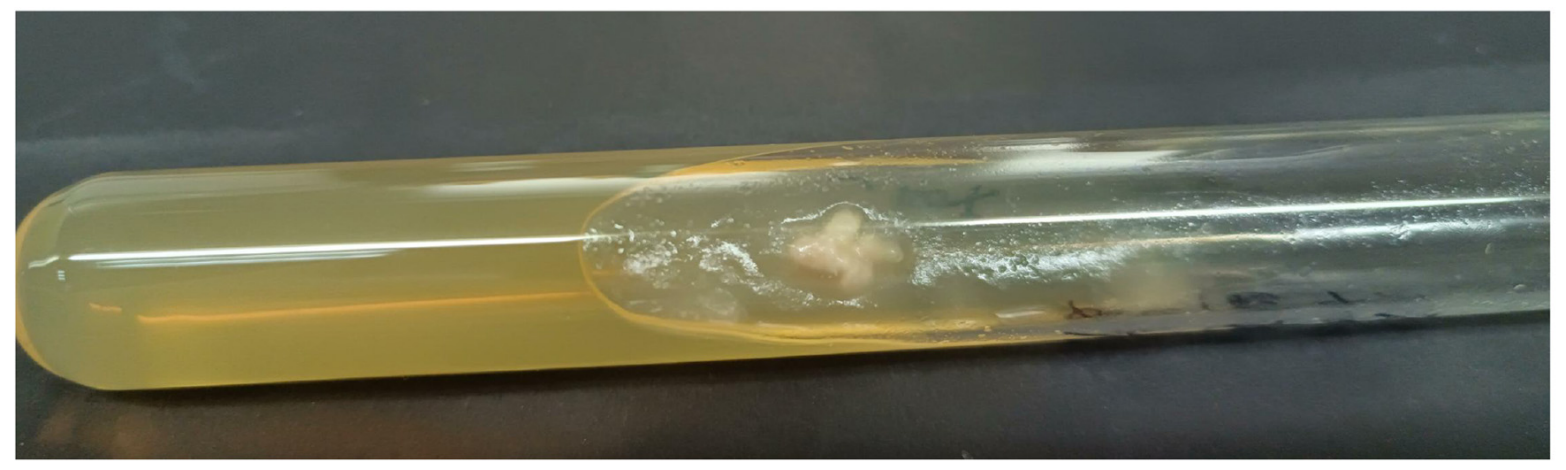
*Coccidioides* colonies after 9 days of culture on SDA at 25 °C.

Samples were collected by extrusion scraping using a sterile lancet. Direct microscopic examination revealed hyphae and thick-walled fungal spherules containing endospores, suggestive of coccidioidomycosis (Figures 3A, B). We immediately sent the samples to the Nordkapp Clinical Laboratory in Hangzhou for metagenomic next-generation sequencing (mNGS). The following day, mNGS analysis strongly indicated the presence of *Coccidioides posadasii*. Given that *C. posadasii* is classified as a high-pathogenicity fungal pathogen under Risk Group 2 by the National Health Commission of the People's Republic of China, The patient was promptly transferred to a single-occupancy room. The room was disinfected daily, and the patient's gowns, bed sheets, and pillowcases were specifically laundered and disinfected. A biopsy specimen of the back lesion was obtained. Staining with hematoxylin and eosin revealed mild hyperkeratosis and epidermal hyperplasia, along with focal aggregates of epithelioid cells and multinucleated cells in the dermis, accompanied by an inflammatory infiltrate composed of lymphocytes, plasma cells, and a small number of eosinophils, and Periodic Acid-Schiff staining showed the presence of multiple fungal spherules contained numerous endospores (Figure 4). Fungal culture was performed by the Mycology Laboratory at the Institute of Dermatology, Chinese Academy of Medical Sciences (Nanjing). After 9 days of incubation at 25 °C, raised, moist, and waxy colonies with sparse aerial hyphae were observed on Sabouraud agar (Figure 5). Microscopic examination revealed hyaline, septate hyphae and abundant barrel-shaped arthroconidia. The above-mentioned institution performed ITS sequencing using primers ITS1F (TCCGTAGGTGAACCTGCGG) and ITS4R (TCCTCCGCTTATTGATATGC). The obtained gene sequence from Sanger sequencing was subjected to BLAST analysis against the NCBI database, which revealed 100% identity with accession number MH862949.1. The causative agent was thus inferred to be *Coccidioides posadasii* (GenBank PZ311798). The isolate was definitively identified as *C. posadasii* by Internal Transcribed Spacer (ITS) sequencing. Furthermore, the institution subsequently performed *in vitro* antifungal susceptibility testing on the isolate using the broth microdilution method. The minimum inhibitory concentrations (MICs) were as follows: 5-Fluorocytosine >64 μg/mL; Posaconazole 0.25 μg/mL; Voriconazole 0.06 μg/mL; Itraconazole 0.5 μg/mL; Amphotericin B 0.5 μg/mL; Fluconazole 8 μg/mL. Following confirmation of the cutaneous coccidioidomycosis diagnosis, and based on the guideline recommendations, the antifungal susceptibility testing results, and the patient′s prior treatment response, the patient was treated with oral fluconazole at a dose of 400 mg daily and was discharged on November 15, 2021. At the same time, the patient was instructed to perform disinfection of their daily living environment.

During the treatment course, the alanine aminotransferase (ALT) levels continued to rise, measuring 131 U/L (November 27, 2021), 132 U/L (December 18, 2021), 191 U/L (January 15, 2022), and 270 U/L (January 29, 2022). No clinical improvement was observed, and direct microscopic examination remained positive. To prevent potential endospore release during direct microscopic examination, after preparing the smear and applying a coverslip, the edges were promptly sealed with a fast-drying organic mounting medium (Ningbo Tongsheng Biotechnology Co., Ltd.). Suspecting that the elevated liver enzymes might be induced by fluconazole, its administration was discontinued. On February 21, 2022, the patient was admitted in preparation for treatment with liposomal amphotericin B. Laboratory tests showed an ALT level of 195 U/L, a glycylproline dipeptidyl aminopeptidase level of 184 U/L (normal range: 44–116 U/L), a low-density lipoprotein level of 3.96 mmol/L (normal range: 1.55–3.36 mmol/L), and a triglyceride level of 3.84 mmol/L (normal range: 0.34–1.7 mmol/L). An initial infusion dose of 150 mg of liposomal amphotericin B was administered. However, during the infusion, the patient developed fever, chills, general malaise, and headache, which were intolerable, leading to discontinuation of the medication. The patient was discharged on March 3, 2022, with the recommendation to undergo lipid-lowering therapy before resuming fluconazole treatment.

Beginning on April 9, 2022, the patient consistently engaged in self-directed weight loss and received lipid-lowering therapy. During this period, the alanine aminotransferase (ALT) levels continued to decline, reaching 35 U/L by October 7, 2025. Over the 3-year period, the patient intermittently took oral fluconazole. During this time, the patient also received oral voriconazole (300 mg twice daily) for 3 months, but this was eventually discontinued due to the adverse reaction of photophobia. Direct microscopic examination results fluctuated between positive and negative, and the skin lesions persisted. On October 28, 2025, the patient underwent a 2-week course of liposomal amphotericin B treatment at an external hospital, which led to significant improvement in the skin lesions. However, direct microscopy still revealed hyphae and spherules without endospores. The patient subsequently continued treatment with fluconazole.

## Discussion

Coccidioidomycosis is currently known to be caused by two dimorphic fungi: *C. immitis* and *C. posadasii*. *Coccidioides* thrives in regions with low annual rainfall (12–50 cm), mild winters that rarely freeze, and alkaline soils (3). *C. immitis* is predominantly endemic to arid and semi-arid areas in the southwestern United States and northern Mexico. In contrast, *C. posadasii* is mainly found in northern Mexico, the southern United States (particularly Texas), and desert regions of certain South American countries. In the United States, more than 95% of coccidioidomycosis cases are reported from California and Arizona. The incidence rate reaches 101 per 100,000 population in Arizona and 18.2 per 100,000 in California. Epidemiological assessment using coccidioidin skin testing in South America indicates prevalence rates as follows: Colombia, 3%−13%; Venezuela, 11%−46%; Brazil, 26%; and Paraguay, 15%−44% (5). These figures may vary due to factors such as changes in rainfall patterns and shifts in susceptible populations. Currently, there are no population-based incidence data for coccidioidomycosis in China. Coccidioidomycosis is rarely reported in China. From 1958 to 2021, only 47 cases were reported, most of whom had a history of travel to endemic areas. Clinicians generally have limited awareness of the disease, leading to high rates of misdiagnosis and underdiagnosis (6, 7). Therefore, attention should also be paid to the fact that, owing to limited infrastructure and insufficient awareness of this emerging threat, there may be many undiagnosed or misdiagnosed latent cases.

*Coccidioides* species are fungi belonging to the division Ascomycota, class Eurotiomycetes, order Onygenales (8, 9). This order comprises several dimorphic human pathogens capable of causing invasive disease in immunocompetent hosts, including *Histoplasma capsulatum, Paracoccidioides* spp., and *Blastomyces* spp (10). *C. immitis* and *C. posadasii* are morphologically indistinguishable, and their predicted proteins share over 90% homology (8). Although serologic tests cannot differentiate between the two species, they can be distinguished by genetic polymorphisms, and some differences in growth characteristics have also been reported (11, 12). *C. posadasii* has a larger effective population size and exhibits greater genetic diversity than *C. immitis* (13).

As a dimorphic fungus, *Coccidioides* alternates between a saprobic phase (mycelial form) and a parasitic phase (spherule form). In the environment, the fungus persists in the mycelial phase within soil, where it functions as a saprotroph, deriving nutrients from decaying organic matter (14, 15). During this phase, it produces asexual spores called arthroconidia. When the soil is disturbed, these arthroconidia become airborne and, if inhaled by a mammalian host, undergo a morphological transition to the parasitic phase. In this state, the fungus derives nourishment from host tissues and forms endospores. These endospores mature into spherules, each containing numerous endospores. Upon rupture of mature spherules, the released endospores disseminate within the host, perpetuating the parasitic cycle, and may also re-enter the environment to resume the saprobic phase in soil (16). Endospores can be disseminated hematogenously or via the lymphatic system to virtually any organ, where they establish new spherules and may lead to disseminated disease (17). Compared with other pathogenic fungi, *Coccidioides* exhibits higher transmissibility and pathogenicity, making it the only fungus included in the U.S. government's list of potential dangerous biological weapon pathogens (18).

Accidental exposure to cultures of *Coccidioides* is a significant route of infection. Even laboratories in non-endemic regions may culture *Coccidioides* from specimens obtained from imported cases; therefore, microbiology laboratories should remain vigilant. The 2016 IDSA guidelines dedicate a section to the prevention of laboratory exposure and post-exposure management protocols (19). Key preventive measures include: all filamentous fungal cultures must only be opened within a biological safety cabinet, and clinicians should promptly inform microbiology laboratory personnel when coccidioidomycosis is suspected. In the event of a laboratory exposure, personnel should evacuate immediately. The potentially contaminated area should be cordoned off prior to disinfection and sterilization procedures. All potentially exposed individuals should be registered, baseline blood samples collected for antibody testing, and coccidioidin skin testing performed. For non-pregnant individuals who have been exposed, prophylactic treatment with fluconazole 400 mg once daily for 6 weeks is recommended, along with observation and follow-up for at least 6 weeks.

Culture and histopathological assessment remain the definitive means of diagnosing coccidioidomycosis (2), while alternative methods can provide earlier and, in some cases, more sensitive approaches for establishing a presumptive diagnosis. Serological antibody testing for coccidioidomycosis is typically performed using immunodiffusion or, more commonly, enzyme immunoassay (EIA) for the detection of IgM and IgG antibodies (3). However, antibody-based testing is routinely conducted only in endemic regions; many clinical laboratories in non-endemic areas—including China—do not offer such assays, making the diagnosis of imported cases particularly challenging.

mNGS is an emerging molecular diagnostic technique that offers multiple advantages over traditional methods. It enables the rapid detection of nearly all known pathogens in a single assay, with high sensitivity and specificity (20–23). mNGS is especially recommended for infections caused by novel, rare, fastidious, or mixed pathogens (24). Importantly, it also bypasses the limitations associated with culture-based identification of highly pathogenic organisms, which are often restricted to biosafety level 3 or higher laboratories. To prevent environmental release and dissemination of infectious spores, we recommend that the patient be placed in a single-occupancy room, with daily environmental disinfection. All linens, clothing, and waste materials should be handled and packaged separately.

Once infection occurs, *Coccidioides* typically presents in tissue as spherules containing endospores. The spherules measure approximately 60–100 μm in diameter and enclose smaller endospores (4–10 μm), which are responsible for dissemination of the infection (25–28). Although hyphal forms have occasionally been reported in tissue, such findings are rare. In the present case, direct microscopic examination of the clinical specimen revealed not only the characteristic spherules containing endospores but also the presence of hyphae. Direct microscopy therefore remains a valuable diagnostic tool, particularly in regions where *Coccidioides* is classified as a highly pathogenic organism and fungal culture is restricted. However, its accurate interpretation requires experienced personnel, which poses a challenge in non-endemic areas. Smear preparation should be performed in a biosafety cabinet, and slides must be sealed with mounting medium to ensure safety during examination.

Approximately 60% of human exposures result in asymptomatic infection, whereas the remaining 40% lead to symptomatic illness, ranging from a self-limited pulmonary infection resembling influenza to, in rare cases, life-threatening disseminated disease (29). *Coccidioides* respiratory infections are a common cause of community-acquired pneumonia among both healthy individuals and immunocompromised patients residing in endemic regions of the southwestern United States (30). In our case, the initial pulmonary infection was likely acquired in Arizona, United States, due to *Coccidioides* exposure, with subsequent dissemination to the skin resulting in cutaneous coccidioidomycosis.

Early diagnosis and treatment can lead to improved clinical outcomes, reduced patient anxiety, and fewer invasive procedures or treatments (31). The latest IDSA Clinical Practice Guideline for the Treatment of Coccidioidomycosis recommends itraconazole 200 mg twice daily or fluconazole 400–800 mg daily for a minimum of 6 to 12 months for the management of soft tissue infections, regardless of the antifungal agent selected (19). In cases of severe or refractory coccidioidomycosis, amphotericin B formulations are typically the treatment of choice (32). Among these, lipid-based formulations are preferred for systemic therapy due to their potential for enhanced efficacy and reduced nephrotoxicity compared to the deoxycholate formulation (33, 34). Fluconazole is available in both oral and parenteral formulations and is unique among azoles because it is primarily metabolized by the kidneys, unlike other agents in this class, which undergo hepatic metabolism (35). Nevertheless, transient elevations in transaminases may be observed with fluconazole administration (35, 36). In patients with invasive fungal infections, the presence of pre-existing liver disease poses additional challenges to clinical management. Notably, alterations in the pharmacokinetics of antifungal drugs and concerns regarding their tolerability in this population are critical considerations (37). The elevation in hepatic enzyme levels observed in our patient was attributed to nonalcoholic fatty liver disease (NAFLD), a condition whose increasing prevalence and severity are closely linked to the obesity epidemic (38). Concerns regarding potential hepatotoxicity led to the administration of suboptimal doses of fluconazole and an unstable treatment course, which ultimately resulted in poor therapeutic outcomes. Subsequent lifestyle modifications aimed at weight reduction, combined with lipid-lowering therapy, successfully normalized liver enzyme levels. Concurrently, the introduction of a short course of liposomal amphotericin B led to a marked improvement in the patient's skin lesions.

Additionally, the difficulty of treatment may be attributed to the inherent virulence of the fungus and/or the fact that its *in vivo* form consists of spherules—large structures that are resistant to phagocytosis and possess antiphagocytic surface properties (39). Furthermore, *coccidioidal* parenchymal lesions are characteristically suppurative and often associated with granuloma formation, features that may impede the penetration of antifungal agents (40).

Except for the northwestern region, most parts of China are not suitable ecological niches for *Coccidioides* species. Most reported cases in non-endemic areas worldwide have a history of travel or residence in endemic regions. However, a survey conducted in China showed that up to 80% of cases had no clear history of exposure to endemic areas (41). Moreover, *Coccidioides* species have been isolated from soil in the Washington area, which is far from the endemic regions in the United States (42). Therefore, it is necessary to implement good biosafety precautions to prevent potential community transmission. In the present case, we actively carried out environmental disinfection in the hospital and advised the patient to perform environmental disinfection in their living quarters.

Delays in the diagnosis of coccidioidomycosis have been shown to be costly (43). In the present case, the patient received treatment for pneumonia at another hospital but remained undiagnosed for a prolonged period, allowing the pathogen to disseminate to the skin and resulting in a complicated, refractory case of coccidioidomycosis.

In conclusion, the diagnosis and treatment of imported highly pathogenic fungal infections resembling coccidioidomycosis in non-endemic regions pose a substantial clinical challenge. Although NGS is relatively expensive, it offers a rapid and effective diagnostic approach. Direct microscopic examination, while requiring specialized personnel for execution, is rapid and inexpensive. The combination of these two methods represents an effective strategy for diagnosing such diseases, particularly in healthcare facilities lacking access to appropriate biosafety laboratories. For patients with underlying conditions that may compromise tolerance to antifungal therapy, a careful balance of these complex factors is essential to achieve optimal treatment outcomes.

## Data Availability

The original contributions presented in the study are included in the article/supplementary material, further inquiries can be directed to the corresponding author.
